# In Vivo Functional and Structural Retinal Preservation by Combined Administration of Citicoline and Coenzyme Q10 in a Murine Model of Ocular Hypertension

**DOI:** 10.3390/ijms27021012

**Published:** 2026-01-20

**Authors:** Jose A. Matamoros, Elena Salobrar-García, Juan J. Salazar, Inés López-Cuenca, Lorena Elvira-Hurtado, Miguel A. Martínez, Sara Rubio-Casado, Víctor Paleo-García, Rosa de Hoz, José M. Ramírez, Pedro de la Villa, Jose A. Fernández-Albarral, Ana I. Ramirez

**Affiliations:** 1 Ramon Castroviejo Institute for Ophthalmic Research, Complutense University of Madrid (ROR 02p0gd045), 28040 Madrid, Spain; jomatamo@ucm.es (J.A.M.); elenasalobrar@med.ucm.es (E.S.-G.); jjsalazar@med.ucm.es (J.J.S.); inelopez@ucm.es (I.L.-C.); marelvir@ucm.es (L.E.-H.); miguma19@ucm.es (M.A.M.); srubio02@ucm.es (S.R.-C.); rdehoz@med.ucm.es (R.d.H.); ramirezs@med.ucm.es (J.M.R.); 2 Health Research Institute of the Hospital Clínico San Carlos (IdISSC) (ROR 014v12a39), 28040 Madrid, Spain; 3 Department of Immunology, Ophthalmology and ENT, Faculty of Optics and Optometry, Complutense University of Madrid, 28040 Madrid, Spain; 4 Department of Immunology, Ophthalmology and ORL, Faculty of Medicine, Universidad Complutense de Madrid, 28040 Madrid, Spain; 5 Department of Physiology, Faculty of Medicine, Universidad Complutense de Madrid, 28040 Madrid, Spain; vipaleo@ucm.es; 6 Department of Systems Biology, University of Alcala, 28801 Madrid, Spain; pedro.villa@uah.es; 7 Visual Neurophysiology Group-IRYCIS, 28034 Madrid, Spain

**Keywords:** Glaucoma, ocular hypertension, neuroprotection, OCT, ERG, Citicoline, Coenzyme Q10

## Abstract

This study evaluated the early structural and functional effects of combined citicoline and coenzyme Q10 (CoQ10) (CitiQ10) treatment in a laser-induced ocular hypertension (OHT) model in Swiss albino mice, focusing on retinal inflammation and neuroprotection. Sixty male CD-1 mice were assigned to four groups: vehicle, CitiQ10, OHT, and OHT + CitiQ10. OHT was induced by laser photocoagulation of limbal and episcleral veins, and CitiQ10 was administered orally starting 15 days before induction. Intraocular pressure (IOP) was measured by rebound tonometry, retinal structure was assessed by spectral domain optical coherence tomography (SD-OCT), and function was evaluated using full-field electroretinography (ffERG). At 3 days post-induction, OHT eyes exhibited significant retinal nerve fiber layer (RNFL) thickening, increased vitreous particles, and early functional impairment, particularly reduced scotopic b-wave and oscillatory potentials. CitiQ10 treatment mitigated these changes, reducing vitreous particles, moderating RNFL alterations, and not exhibiting significant changes in ERG amplitudes. At 7 days post-induction, structural and functional deficits persisted but were less pronounced in treated eyes. These findings suggest that CitiQ10 treatment may attenuate early retinal damage in glaucoma, with OCT and ffERG serving as reliable monitoring tools, supporting the therapeutic potential of this approach in early stage disease.

## 1. Introduction

Glaucoma is one of the leading causes of irreversible blindness worldwide [1,2,3]. In 2020, approximately 76 million people were affected, and this number is expected to rise to 112 million by 2040 [1,3,4,5]. Due to its slow progression and compensatory capacity of central visual pathways, glaucoma is often diagnosed at advanced stages [2,6]. For this reason, optical coherence tomography (OCT) and ocular electrophysiology are key tools in the diagnosis and monitoring of glaucoma [7,8]. OCT provides high-resolution imaging of retinal structures and shows strong diagnostic performance, with sensitivities of 60–98% and specificities of 80–95% for detecting glaucomatous damage through peripapillary retinal nerve fiber layer (RNFL) measurements [9]. It also outperforms visual field testing in detecting early progression, identifying changes in 38.9% of cases compared with 18.7% detected by perimetry [9,10]. In humans, progressive degeneration of retinal ganglion cells (RGCs) and their axons typically manifests as RNFL thinning, especially in the superior and inferior sectors of the optic disk regions commonly affected in the early stages of glaucoma [11].

Glaucoma also involves a neuroinflammatory component mediated by glial cells. This inflammation, often subclinical and chronic, may initially serve a protective role but can become detrimental if dysregulated [12]. Advances in OCT have enabled the noninvasive detection of inflammatory changes through quantification of vitreous particles [13,14], which correspond to hyalocytes, macrophage-like cells that regulate the vitreous immune environment [15]. The vitreous matrix facilitates rapid diffusion of these molecules, allowing hyalocytes to respond swiftly to damage signals [16]. Under normal conditions, hyalocytes are few and small [17], but upon activation they enlarge, migrate toward injury sites, and increase their reflectivity on OCT [13,14,18]. High densities of small hyalocytes have been observed on the ciliary body surface in guinea pigs [19] and healthy rats [20], suggesting a potential migratory route into the vitreous. In mice, hyperreflective particles near the optic nerve head and vitreous base correspond to Iba-1 + hyalocytes, indicating that these cells may act as early sensors of retinal damage and may represent potential biomarkers of inflammatory activity in glaucoma [18].

Although glaucoma primarily affects RGCs, other retinal neurons, including those in the inner and outer nuclear layers (INL and ONL), can also be compromised [21,22,23]. Such changes have been demonstrated in vivo using full-field electroretinography (ffERG), which provides objective measurements of retinal function [24,25]. Alterations in ffERG components, including the scotopic threshold response (STR), a-wave, and b-wave, linked to RGCs, photoreceptors, and bipolar cells, respectively, have been reported in both patients and experimental models of glaucoma [25,26,27].

Electroretinography outcomes vary depending on the model and retinal component analyzed. In primates with chronic ocular hypertension (OHT), ffERG responses, such as a- and b-waves and oscillatory potentials (OPs), remain relatively stable, suggesting preserved function [28]. However, other studies have reported conflicting results, with some showing reductions in OPs or in a- and b-wave amplitudes, while others found no significant changes. These discrepancies may be attributed to differences in the frequency bands analyzed or the type of stimulus used (scotopic versus photopic) [29]. In rats that experienced induced OHT, significant reductions in scotopic and photopic a- and b-wave amplitudes are observed at advanced stages [30]. Animal models with sustained or transient IOP elevation also show progressive OP decline, which was evident from week 4 and continued to progress, while the a- and b-waves showed significant decreases between 11 and 14 weeks [31]. A laser-induced OHT model and DAB/2J mice also showed significant reductions in scotopic ERG a- and b-waves between weeks 2 and 12, correlating with morphological alterations in INL and ONL neurons [32]. These findings indicate that retinal damage is closely linked to the intensity and duration of IOP elevation.

In this study, we used a unilateral laser-induced OHT model in adult male CD-1 Swiss albino mice to transiently elevate IOP by disrupting aqueous humor outflow. This approach induces sectorial RGC loss and glial activation, replicating key features of human glaucomatous neuropathy [27,33,34,35].

Our previous work using this model demonstrated that CitiQ10 treatment at 3 and 7 days post-induction reduced retinal inflammation and prevented RGC death [36,37]. However, these histological analyses provide only endpoint information and require tissue extraction. In contrast, OCT and ffERG offer complementary, non-invasive, and longitudinal insight into early retinal alterations. OCT enables in vivo detection of dynamic structural responses, such as thinning associated with neuronal loss or thickening linked to inflammation [38,39], while ffERG captures functional deficits in the outer and inner retinal pathways that cannot be inferred from histology alone [40]. Integrating these in vivo technologies allows us to determine whether early anti-inflammatory and neuroprotective effects observed at the cellular level translate into measurable structural and functional changes relevant to clinical monitoring.

Given the clinical relevance of structural (OCT) and functional (ffERG) assessments in glaucoma, we evaluated RNFL thickness at the same time points used to assess the anti-inflammatory (3 days post-induction) and neuroprotective (7 days post-induction) effects of CitiQ10. We also quantified vitreous particles near the optic nerve head as a potential indirect marker of macrophage-related inflammatory activity and assessed early functional changes following OHT induction to determine whether citicoline and CoQ10 could modulate these responses.

## 2. Results

### 2.1. Intraocular Pressure Dynamics Following OHT Induction

**Figure 1:**
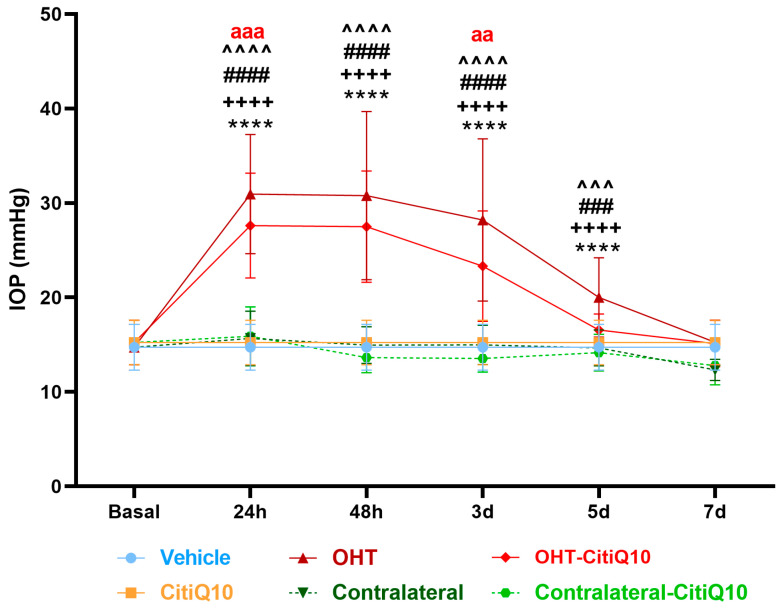
IOP in different study groups after induction of hypertension. Data are mean ± standard deviation (SD). Abbreviations: ocular hypertension (OHT); Citicoline + Coenzyme Q10 (CitiQ10); hours (h); day (d); intraocular pressure (IOP). Statistical significance indicators: **** *p* < 0.0001 vehicle vs. OHT; ++++ *p* < 0.0001 CitiQ10 vs. OHT-CitiQ10; ### *p* < 0.001, #### *p* < 0.0001 OHT vs. contralateral; ^^^ *p* < 0.001, ^^^^ *p* < 0.0001 OHT–CitiQ10 vs. contralateral CitiQ10; aa *p* < 0.01, aaa *p* < 0.001 for OHT vs. OHT-CitiQ10.

In this experimental analysis, IOP was assessed across multiple groups: vehicle, CitiQ10, OHT, contralateral eyes, OHT-CitiQ10, and contralateral-CitiQ10. Measurements were taken at several time points—24 h, 48 h, 3 days, 5 days, and 7 days post-induction. At each time point, eyes subjected to OHT and OHT-CitiQ10 consistently exhibited elevated IOP compared to their respective controls (vehicle and CitiQ10) and contralateral eyes, with statistically significant differences observed in most comparisons (*p* < 0.0001), except at specific time points where *p*-values ranged between <0.001 and <0.01 (Figure 1).

At 7 days post-induction, IOP values in OHT and OHT-CitiQ10 eyes were comparable to those of their respective controls and contralateral eyes. Notably, eyes treated with the CitiQ10 treatment showed significantly lower IOP than untreated OHT eyes at 24 h (*p* < 0.001) and 3 days (*p* < 0.01) post-induction, suggesting a modest hypotensive effect of the treatment during the early phase following OHT induction (Figure 1).

### 2.2. Early Detection of OHT-Induced Alterations Using OCT

Currently, OCT has become an indispensable tool for the diagnosis and monitoring of ocular diseases. In the context of glaucoma, the assessment of the RNFL thickness enables in vivo observation of disease progression, as this layer is susceptible to damage resulting from the gradual degeneration of RGCs.

During in vivo OCT imaging, 5 of the 18 animals in the OHT group could not be evaluated because marked media opacity prevented the acquisition of diagnostic-quality scans. In contrast, within the OHT-CitiQ10 group, only 1 of the 18 animals could not be imaged due to media opacity; in the remaining animals, mild media opacities were observed, but diagnostic OCT images could still be obtained. In all other experimental groups, OCT acquisition was successfully performed in every animal. Consequently, the number of animals for which OCT imaging was available was as follows: vehicle (*n* = 18), OHT (*n* = 13), contralateral (*n* = 18), CitiQ10 (*n* = 18), OHT-CitiQ10 (*n* = 17), and Contralateral-CitiQ10 (*n* = 18).

#### 2.2.1. Early Changes in RNFL Thickness Following OHT Induction and the Modulatory Effect of Combined Citicoline and Coenzyme Q10 Treatment

RNFL thickness centered on the optic disk was analyzed at 3 and 7 days following OHT induction across all experimental groups.

**Figure 2:**
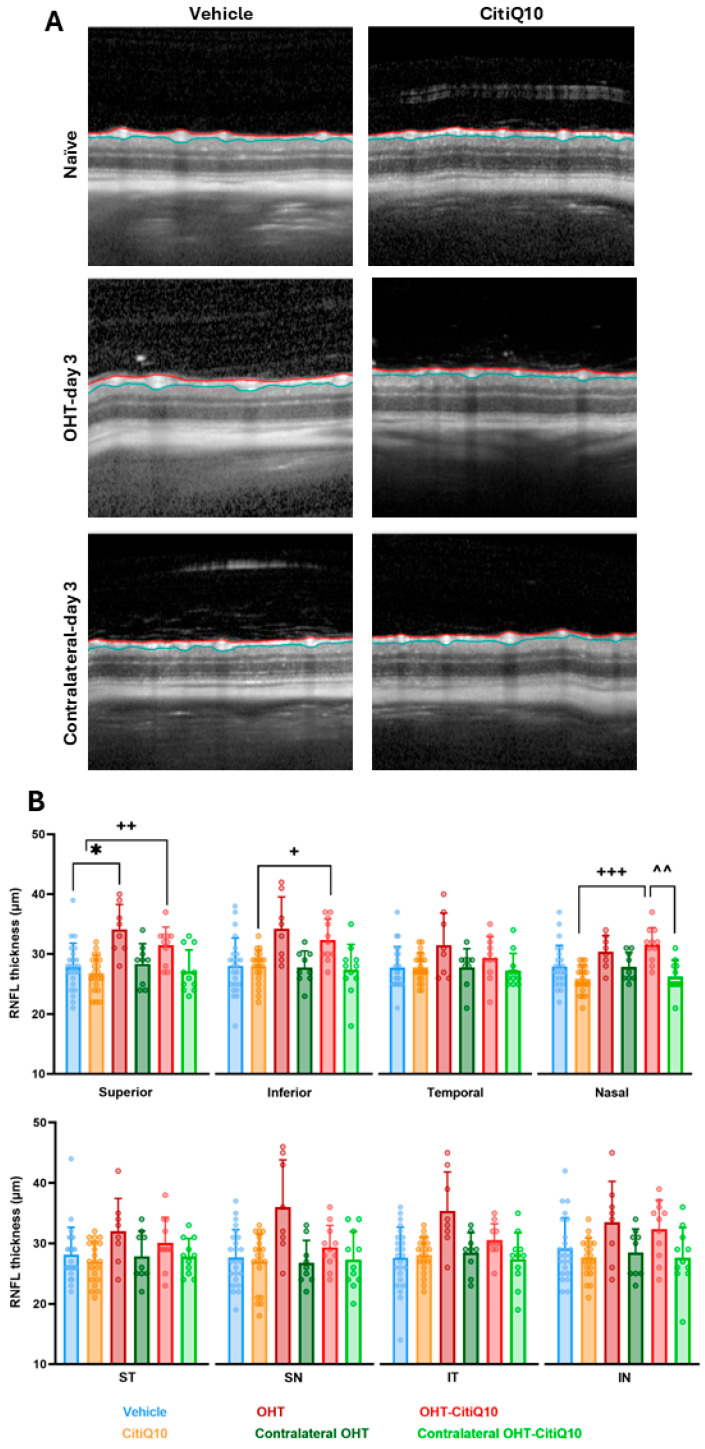
Representative cross-sectional OCT scans (**A**) and retinal nerve fiber layer (RNFL) thickness measured by OCT (**B**) in the different experimental groups at 3 days post-induction. The analyzed sectors were SN: supero-nasal; ST: supero-temporal; IN: infero-nasal; IT: infero-temporal. Statistical significance indicators: * *p* < 0.05 vs. vehicle; + *p* < 0.05; ++ *p* < 0.01; +++ *p* < 0.001 vs. CitiQ10; ^^ *p* < 0.01 OHT vs. Contralateral-CitiQ10. Two-way ANOVA was used. Abbreviations: OCT (optical coherence tomography); OHT (ocular hypertension); Citicoline + Coenzyme Q10 (CitiQ10); retinal nerve fiber layer (RNFL); supero-nasal (SN); supero-temporal (ST); infero-nasal (IN); infero-temporal (IT).

At 3 days post-induction, a statistically significant increase in RNFL thickness was observed in the superior region of the OHT eyes compared to the vehicle group (*p* < 0.05). Although other regions showed a tendency toward increased thickness, these changes did not reach statistical significance. Additionally, a significant increase in RNFL thickness was detected in the OHT-CitiQ10 group compared to their respective control group (CitiQ10) in the superior (*p* < 0.01), inferior (*p* < 0.05), and nasal (*p* < 0.001) sectors, as well as compared to the contralateral eye (Contralateral-CitiQ10) in the nasal region (*p* < 0.01). No significant changes were observed in the remaining sectors (Figure 2).

**Figure 3:**
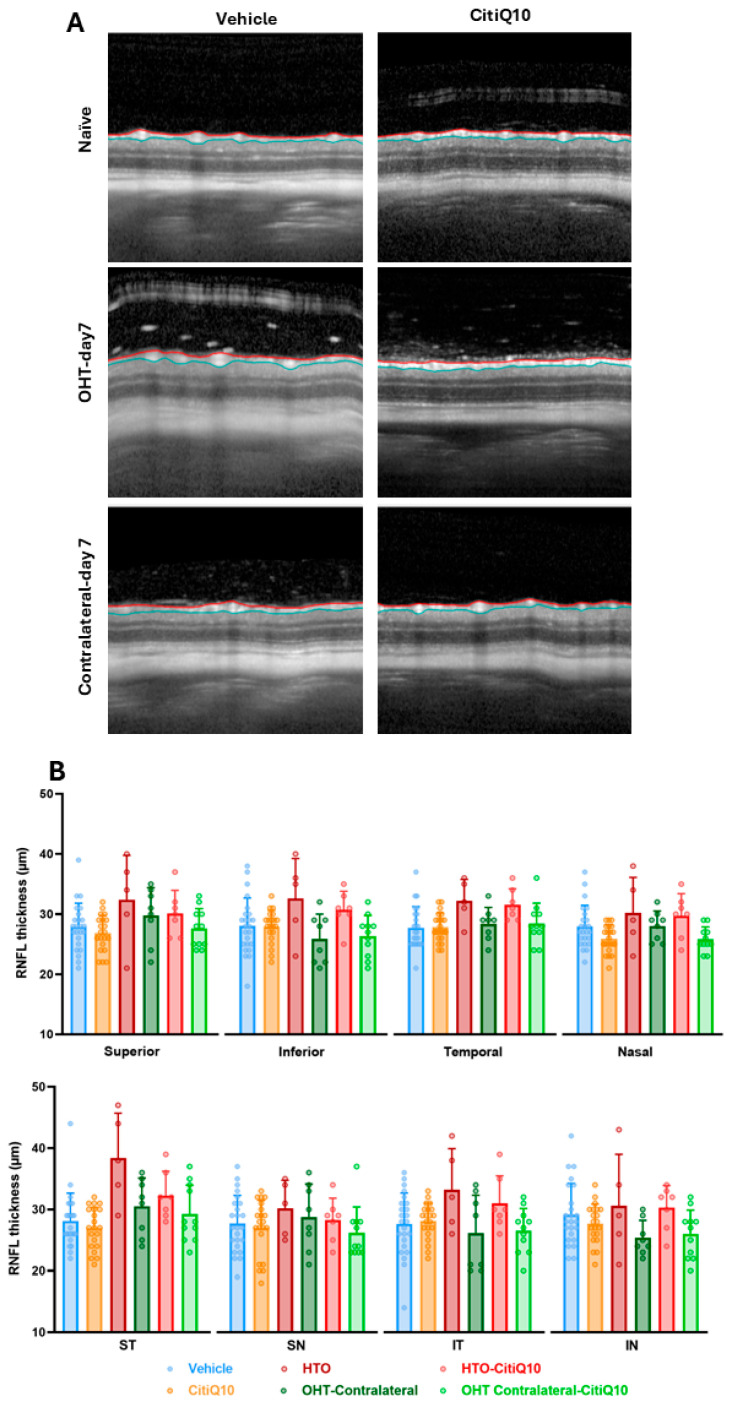
Representative cross-sectional OCT scans (**A**) and retinal nerve fiber layer (RNFL) thickness measured by OCT (**B**) in the different experimental groups at 7 days post-induction. The analyzed sectors were SN: supero-nasal; ST: supero-temporal; IN: infero-nasal; and IT: infero-temporal. Two-way ANOVA was used. Abbreviations: OCT (optical coherence tomography); OHT (ocular hypertension); Citicoline + Coenzyme Q10 (CitiQ10); retinal nerve fiber layer (RNFL); supero-nasal (SN); supero-temporal (ST); infero-nasal (IN); infero-temporal (IT).

At 7 days post-induction, no statistically significant differences were detected among the experimental groups or in any of the analyzed regions. However, a tendency toward increased RNFL thickness was noted in the OHT eyes compared to those treated with the CitiQ10 combination (OHT-CitiQ10) (Figure 3).

#### 2.2.2. OHT Induces Vitreous Particle Accumulation at Early Stages, Which Is Modulated by Early Treatment with Citicoline and Coenzyme Q10

An analysis of the number of vitreous particles in the basal vitreous was conducted at 3 and 7 days post-induction, using OCT images focused on the optic nerve. The presence of these particles may be associated with inflammatory cells, suggesting an underlying inflammatory process—a key factor in diseases such as glaucoma.

**Figure 4:**
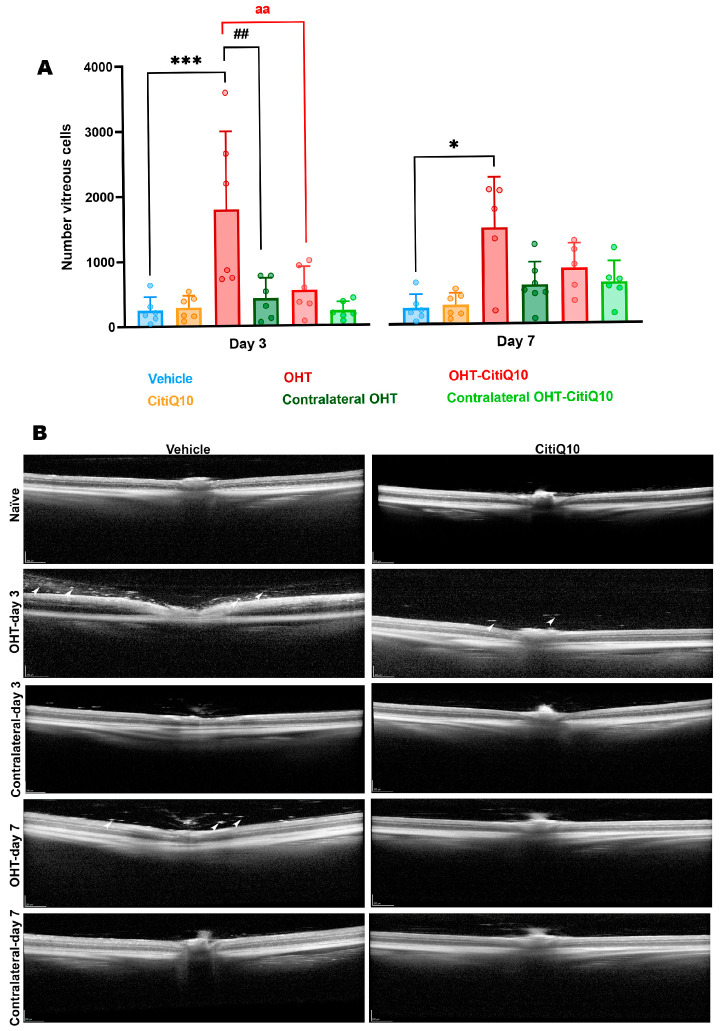
Number of vitreous particles in the different experimental groups at 3 and 7 days post-induction (**A**), along with representative cross-sectional OCT scans at the level of the optic nerve for the analysis of vitreous particles in the same groups and time points (**B**). Statistical significance indicators: * *p* < 0.05, *** *p* < 0.001 vs. vehicle; ## *p* < 0.01 vs. contralateral; aa *p* < 0.01 OHT vs. OHT-CitiQ10. Two-way ANOVA was used. Abbreviations: OHT (ocular hypertension); Citicoline + Coenzyme Q10 (CitiQ10). Arrows indicate vitreous particles. Scale bar 200 µm.

At both 3 and 7 days post-induction, a significant increase in the number of vitreous particles was quantified in OHT eyes compared to the vehicle group (*p* < 0.001 at 3 days post-induction; *p* < 0.05 at 7 days post-induction) (Figure 4A,B). Furthermore, at 3 days post-induction—when the particle count was highest—a significant difference was also observed between OHT eyes and their contralateral eyes (*p* < 0.01) (Figure 4A,B). Comparison between OHT eyes and those treated with CitiQ10 (OHT-CitiQ10) revealed a significantly lower number of vitreous particles in the CitiQ10 group (*p* < 0.01) at 3 days post-induction (Figure 4A,B).

#### 2.2.3. The Number of Vitreous Particles Increases with Elevated IOP, a Response That Is Not Observed in Eyes Treated with Citicoline and Coenzyme Q10

The correlation between the maximum IOP and the number of vitreous particles was analyzed in the untreated groups and the CitiQ10-treated groups.

**Figure 5:**
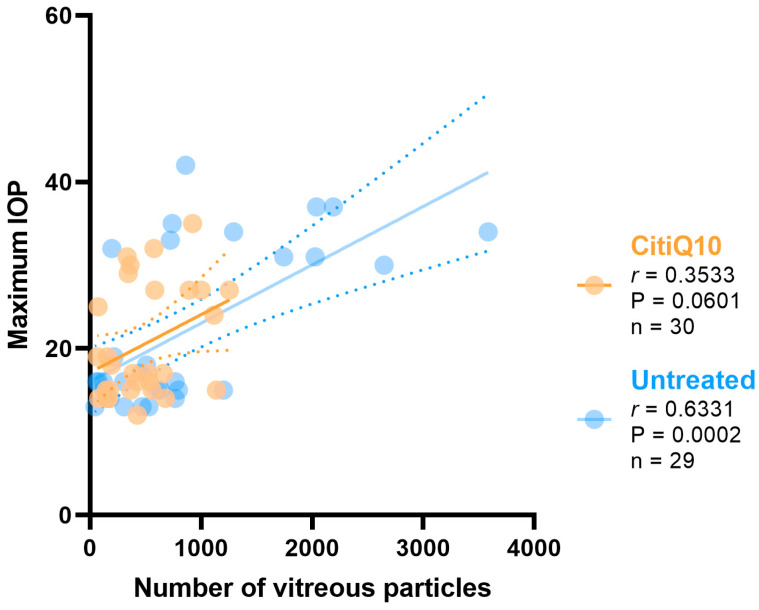
Pearson’s correlation coefficient (r) analysis between the number of vitreous particles and maximum IOP in the untreated eyes (vehicle and OHT groups) and the treated (CitiQ10 and OHT- CitiQ10 groups). Abbreviations: intraocular pressure (IOP); Citicoline + Coenzyme Q10 (CitiQ10); Pearson’s correlation coefficient (r); *p*-value (P); number of samples analyzed (*n*).

The IOP value has a positive strong correlation with the number of vitreous particles in the untreated groups (r = 0.6331, *p* > 0.001); however, no correlation was found in the CitiQ10-treated eyes (r = 0.3533, *p* = 0.0601) (Figure 5).

### 2.3. ffERG Reveals Early Dysfunction in Outer Retinal Activity Post-OHT Induction

The functional activity of cells located in the outer retinal layers was assessed using ffERG. Although these cells are not directly affected by glaucoma, as RGCs are, they may undergo secondary alterations. Like OCT, this non-invasive technique provides real-time insights into disease progression and is valuable for monitoring. Changes in ffERG recordings were analyzed across the experimental groups at 3 and 7 days post-induction, along with the potential impact of combined treatment with CitiQ10.

#### 2.3.1. Early Retinal Damage at 3 Days Post-Induction Is Reflected by Reduced Scotopic b-Wave and Oscillatory Potentials (OPs), with Partial Protection by CitiQ10

**Figure 6:**
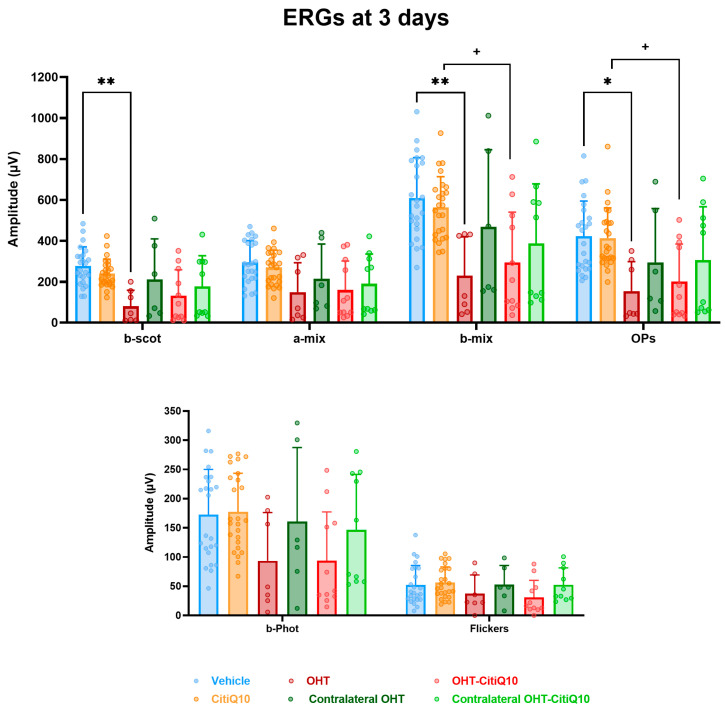
Maximum amplitudes of the different waves comprising the ERG across experimental groups 3 days post-induction. Values are presented as mean ± standard deviation. Statistical significance indicators: * *p* < 0.05; ** *p* < 0.01 vs. vehicle; + *p* < 0.05 vs. CitiQ10. Abbreviations: ERG (electroretinogram), OHT (ocular hypertension), Citicoline + Coenzyme Q10 (CitiQ10), b-scot (scotopic b-wave), a-mix (mixed a-wave), b-mix (mixed b-wave), OPs (oscillatory potentials), b-phot (photopic b-wave), and microvolts (μV).

At 3 days post-induction, analysis of the mean amplitude of the different waves comprising the ffERG revealed a significant reduction in the scotopic b-wave (*p* < 0.01), mixed b-wave (*p* < 0.01), and oscillatory potentials (*p* < 0.05) in OHT eyes compared to vehicle eyes (Figure 6). However, in OHT-CitiQ10 animal eyes, this significant reduction was observed only in the mixed b-wave and oscillatory potentials (*p* < 0.05) when compared to their respective control eyes (CitiQ10) (Figure 6).

**Figure 7:**
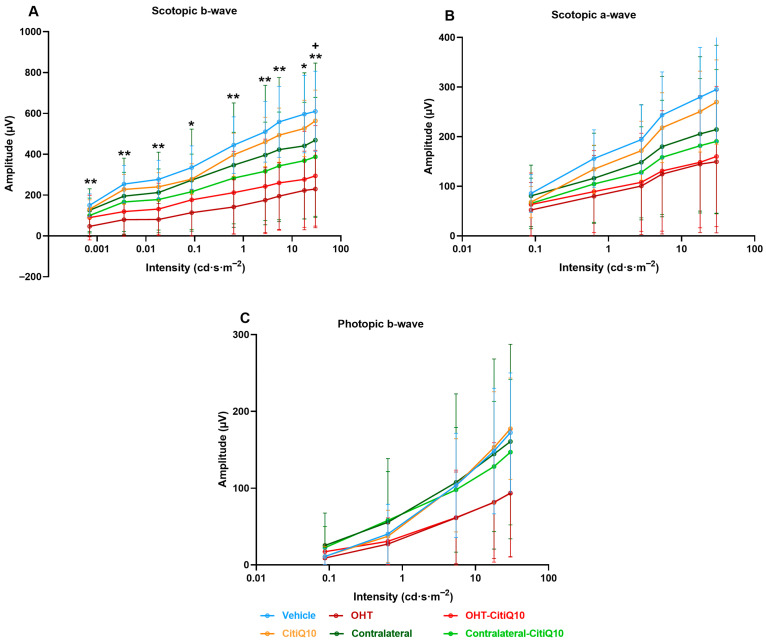
Intensity–amplitude graph of the scotopic b-wave (**A**), scotopic a-wave (**B**), and photopic b-wave (**C**) across experimental groups 3 days post-induction. Each line represents one experimental group, with mean amplitude and standard deviation (SD) shown at each stimulus intensity. Statistical significance indicators: * *p* < 0.05; ** *p* < 0.01 vs. vehicle; + *p* < 0.05 vs. CitiQ10. Abbreviations: SD (standard deviation); OHT (ocular hypertension); Citicoline + Coenzyme Q10 (CitiQ10); microvolts (μV); candela (cd); seconds (s); meters (m).

Analysis of the variation in **scotopic b-wave** amplitude showed a reduction across all stimulus intensities when comparing the control groups (vehicle and CitiQ10) with their contralateral and OHT eyes. This reduction became statistically significant in OHT-untreated eyes at all tested intensities (*p* < 0.05 for 0.087 and 17.92 cd·s·m^−2^; *p* < 0.01 for the remaining intensities). However, in OHT eyes treated with the CitiQ10 combination (OHT-CitiQ10), a significant difference compared to their control (CitiQ10) was observed only at the highest intensity (*p* < 0.05) (Figure 7A).

In the case of the **scotopic a-wave**, a similar decreasing trend was observed, although it was more pronounced in OHT groups (OHT and OHT-CitiQ10), followed by contralateral eyes of these groups compared to their respective controls (vehicle and CitiQ10). No statistically significant differences were found at any of the tested intensities (Figure 7B).

In the analysis of the **photopic b-wave**, a similar pattern to that observed in the scotopic a- and b-waves was found. However, in this case, the contralateral eyes (Contralateral-OHT and contralateral OHT-CitiQ10) showed a response very similar to that of the control groups, unlike the OHT eyes (OHT and OHT-CitiQ10), which exhibited a consistent reduction in amplitude across all tested light intensities (Figure 7C).

#### 2.3.2. Global Reduction in ERG Wave Amplitudes Following OHT Is Partially Prevented by CitiQ10 Treatment at 7 Days Post-Induction

**Figure 8:**
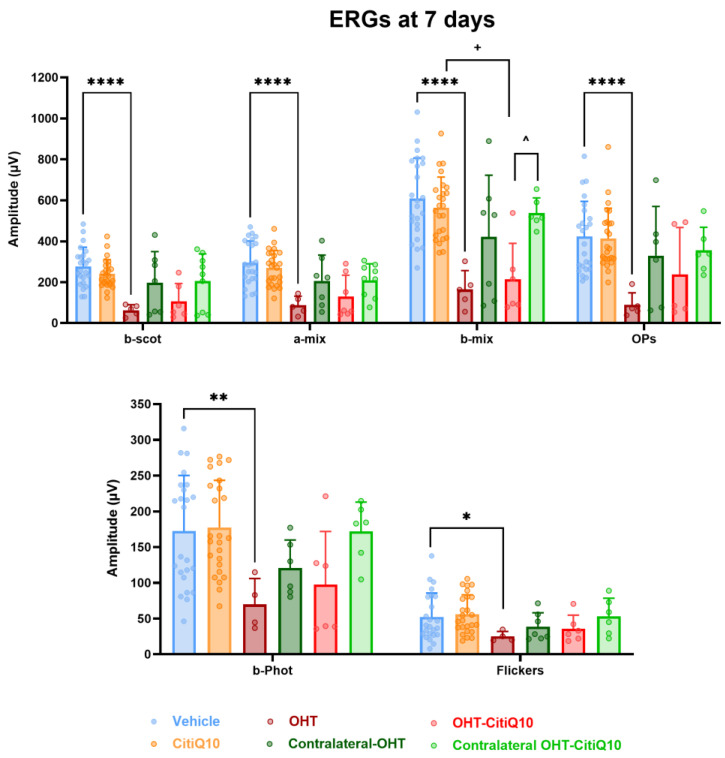
Maximum amplitudes of the different waves comprising the ERG across experimental groups 7 days post-induction. Values are presented as mean ± standard deviation. Statistical significance indicators: * *p* < 0.05; ** *p* < 0.01; **** *p* < 0.0001 vs. vehicle; + *p* < 0.05 vs. CitiQ10; ^ *p* < 0.05 OHT-CitiQ10 vs. Contralateral-CitiQ10. Abbreviations: ERG (electroretinogram); OHT (ocular hypertension); Citicoline + Coenzyme Q10 (CitiQ10); b-scot (scotopic b-wave); a-mix (mixed a-wave), b-mix (mixed b-wave); OPs (oscillatory potentials); b-phot (photopic b-wave); microvolts (μV).

At 7 days post-induction, analysis of the highest amplitudes of characteristic ERG waves revealed a significant reduction in all parameters in OHT eyes compared to the vehicle group (*p* < 0.0001 for scotopic b-wave, mixed response (a and b), and OPs; *p* < 0.05 for photopic b-wave and flickers). However, this significant reduction (*p* < 0.05) was only observed in OHT-CitiQ10 eyes in the b-wave amplitude of the mixed cone and rod response when compared to their respective CitiQ10 control and contralateral OHT-CitiQ10 eyes (Figure 8).

**Figure 9:**
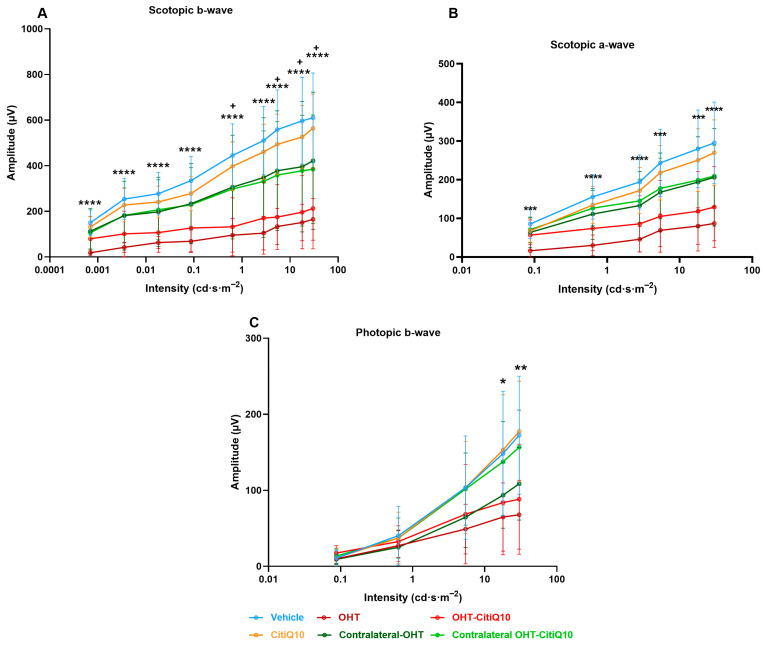
Intensity–amplitude graph of the scotopic b-wave (**A**), scotopic a-wave (**B**), and photopic b-wave (**C**) across experimental groups 7 days post-induction. Each line represents one experimental group, with mean amplitude and standard deviation (SD) shown at each stimulus intensity. Statistical significance indicators. Statistical significance indicators: **** *p* < 0.0001; *** *p* < 0.001; ** *p* < 0.01; * *p* < 0.05 vs. vehicle, + *p* < 0.05 vs. CitiQ10. Abbreviations: SD (standard deviation); OHT (ocular hypertension); Citicoline + Coenzyme Q10 (CitiQ10); microvolts (μV); candela (cd); seconds (s); meters (m).

The analysis of **scotopic b-wave** amplitude variation revealed a consistent reduction across all stimulus intensities in both OHT and contralateral eyes compared to their respective control groups (vehicle and CitiQ10). This decrease was statistically significant at all tested intensities in OHT eyes (*p* < 0.0001), but only at specific intensities in OHT-CitiQ10 eyes (*p* < 0.05 at 0.6342, 5.40575, 17.92, and 30 cd·s·m^−2^) (Figure 9A). Regarding the **scotopic a-wave**, a significant reduction in amplitude was also observed at all stimulus intensities in OHT eyes compared to the vehicle control group (*p* < 0.001 at 0.0871150, 5.40575, and 17.92 cd·s·m^−2^; *p* < 0.0001 at the remaining intensities). However, in OHT-CitiQ10 eyes, no significant differences were observed when compared to their respective CitiQ10 control group. (Figure 9B).

In the analysis of the **photopic b-wave** conducted at 7 days post-induction, amplitude values were comparable across experimental groups at lower stimulus intensities. However, at higher intensities, a significant reduction in amplitude was observed in OHT eyes compared to the vehicle group (*p* < 0.05 at 17.92 cd·s·m^−2^ and *p* < 0.01 at 30 cd·s·m^−2^) (Figure 9C).

## 3. Discussion

This is the first study to simultaneously evaluate structural changes using OCT and functional alterations by ERG to assess the effects of CitiQ10 treatment in a laser-induced OHT model in albino Swiss mice.

Compared with our previous work based solely on histology, the present study provides additional translational insight by showing that early inflammatory and neurodegenerative responses can be monitored in vivo. RNFL thickening and vitreous hyperreflective particles appear to reflect dynamic glial activation, while ERG recordings showed only non-significant tendencies toward functional impairment at these early time points. These findings suggest that OCT may detect subtle structural and inflammatory changes before clear functional deficits become measurable, highlighting the complementary value of OCT and ffERG for early monitoring in experimental glaucoma.

The laser-induced OHT model in albino Swiss mice is well-suited for investigating retinal damage mechanisms associated with elevated IOP and for testing potential neuroprotective therapies [27,41,42].

As in previous studies [36,37], we observed a mild but statistically significant hypotensive effect in OHT eyes treated with the combination therapy at 24 h and 3 days, which has been attributed to the anti-inflammatory properties of the treatment [36,37,43]. However, this decrease was small and remained within a range known to induce retinal stress in this model. Therefore, although IOP lowering may contribute to some early differences, it is unlikely to fully explain the structural and inflammatory changes observed, suggesting that early anti-inflammatory or metabolic effects of CitiQ10 may also play a role.

Conventional glaucoma treatments primarily aim to reduce IOP, the main modifiable risk factor. However, neurodegeneration often progresses despite adequate pressure control [44], highlighting the need for therapies that target additional pathogenic pathways involved in RGC loss. Citicoline and CoQ10 have emerged as promising candidates in this context.

Citicoline, a precursor of membrane phospholipids and acetylcholine, has demonstrated neuroprotective effects in both animal models and clinical studies. These include the preservation of RGCs, reduction in pro-inflammatory cytokines, and improvements in GCL and RNFL thickness [45,46], as well as enhanced retinal and cortical electrophysiological responses [47,48] and visual field performance [49,50].

Although extensively studied, CoQ10 has shown antioxidant and neuroprotective properties in experimental and clinical settings [51,52]. Combinations of citicoline or CoQ10 with other compounds have yielded synergistic protective effects [43,53,54,55]. However, only two studies conducted by our group have demonstrated that the combined administration of citicoline and CoQ10 exerts both anti-inflammatory and neuroprotective effects in this specific glaucoma model [36,37]. This neuroprotective action was evident at day 7 after OHT induction, when CitiQ10-treated eyes showed no RGC loss despite markedly elevated IOP, whereas untreated OHT animals exhibited a clear and well-characterized reduction in RGC survival, consistent with the level of degeneration previously reported in this model at this time point [35,56,57].

Building on these findings, the present study aimed to determine whether such effects could also be detected through structural (OCT) and functional (ERG) assessments at the same time points previously analyzed at 3 and 7 days post-induction. These time points were selected based on prior evidence showing peak retinal inflammation and glial activation at 3 days [58] and significant RGCS loss (approximately 33%) at 7 days post-induction [35,56,57].

### 3.1. Effect of Citicoline and CoQ10 on RNFL Thickness Following OHT Induction

In our study, we observed a significant increase in RNFL thickness at 3 days post-induction in untreated eyes, particularly in the superior sector. This thickening likely reflects an active inflammatory response. Although only 13 of the 18 OHT eyes yielded analyzable OCT scans due to laser-induced media opacity, this sample size remains appropriate for reliable structural analysis in murine OCT studies, which commonly include 6–8 animals per group [59]. Therefore, the reduced number of evaluable scans does not compromise the validity of our findings, although OCT scans in eyes with the most severe opacities could not be captured.

In interpreting this early RNFL thickening, it is important to consider that OCT provides an indirect structural readout and therefore reflects global tissue responses rather than specific cellular events. Even so, the timing of these changes is consistent with the early inflammatory phase previously described in this model [58,60], in which glial activation emerges rapidly after IOP increase. This temporal correspondence suggests that the RNFL thickening detected at 3 days post-induction represents an early inflammatory response rather than structural degeneration.

In eyes treated with CitiQ10 (OHT-CitiQ10), a similar increase in RNFL thickness was observed in the superior, inferior, and nasal sectors, whereas the remaining regions showed values comparable to the controls. It is noteworthy that, among the eighteen animals initially included in this group, only one could not undergo OCT imaging due to media opacity, a lower number than in the OHT group, where images could not be obtained from five animals for the same reason. Taken together, these findings support a modulatory effect of the treatment on early inflammatory responses.

At 7 days post-induction, the thickening tendency persisted in untreated eyes but was less pronounced and did not reach statistical significance. Notably, no prior studies have specifically evaluated RNFL thickness in this experimental model. Our results revealed RNFL thickening at both time points, contrary to the expected thinning associated with axonal loss. A previous study using the same model reported a slight reduction in Brn3a+ RGCs in the temporal region, particularly in its central zone, at 3 days post-induction [56], possibly due to decreased marker expression from cellular dysfunction rather than actual neuronal death. Therefore, structural thinning would not be anticipated at this early stage.

At the cellular level, our histological analysis in previous studies [58,60] confirmed that this early structural change coincides with marked microglial and macroglial activation within the RNFL and ganglion cell complex. Notably, these inflammatory changes were less pronounced in eyes treated with citicoline and CoQ10, supporting the idea that the treatment attenuates the initial glial response to elevated IOP and may help preserve structural integrity as pathology progresses.

Despite clear RGC loss at 7 days post-induction, as reported in our study and others [36,42,56], RNFL thinning was not observed. Instead, residual thickening persisted, although without statistical significance. This may reflect a balance between ongoing axonal degeneration and compensatory glial activation, masking structural loss in OCT measurements.

In treated eyes with citicoline and CoQ10, no RGC loss was detected at 7 days post-induction [36], and inflammation appeared more controlled [37]. These factors may explain the absence of significant RNFL changes compared to controls, reinforcing the dual neuroprotective and anti-inflammatory role of the CitiQ10 treatment.

In this study, we also examined the contralateral eyes of those subjected to OHT induction. Previous works using the same experimental model have shown that contralateral eyes may develop early inflammatory changes secondary to unilateral IOP increase [37,58,61,62], although these occur without RGC loss [36]. In our analysis, RNFL thickness did not differ significantly from controls in either untreated contralateral eyes or those treated with CitiQ10 at 3 and 7 days post-induction. Earlier histological findings in this model indicate that contralateral inflammatory responses are mild at these early stages [58,63,64], which helps contextualize the absence of OCT-detectable structural alterations. Together, these observations suggest that contralateral inflammation remains limited and does not compromise RNFL integrity.

Unlike our findings, most glaucoma models report progressive RNFL thinning in OHT eyes. For instance, laser-induced glaucoma shows RNFL reduction from week 3 [65] and surgical models in rats show RNFL reduction from week 3 [66]. Chronic models using PLGA microspheres show fluctuating RNFL thickness from weeks 2–4, with marked thinning by week 8, especially in superior-temporal and inferior-temporal sectors [67]. Dexamethasone–fibronectin microspheres induce RNFL thinning from week 12 [68].

These studies focus on later stages of neurodegeneration, whereas our work examines early phases (at 3 and 7 days post-induction), when inflammation predominates. Early glial activation may hide initial neuronal loss in OCT measurements, explaining the absence of thinning and presence of RNFL thickening. This aligns with clinical findings in uveitic glaucoma, where active inflammation correlates with increased RNFL thickness [69]. Nonetheless, OCT studies in glaucomatous patients generally show progressive RNFL thinning as axonal damage advances [70].

In addition to these glaucoma models, only a few studies have evaluated the effects of neuroprotective treatments on RNFL thickness using OCT in rodents. Small extracellular vesicles derived from mesenchymal stem cells preserved RNFL thickness at day 21 in a laser-induced hypertensive rat model, whereas untreated eyes showed significant thinning [71]. In a chronic mouse model of sustained OHT induced by circumlimbal suturing, treatment with lipoxins—particularly LXB_4_—attenuated RNFL loss after 12 weeks [72]. Similarly, in a chronic rat model induced by episcleral vein occlusion, a monoclonal antibody against GFAP preserved RNFL thickness in a dose dependent manner across weeks 3 to 10 [73]. Together, these studies indicate that structural protection detectable by OCT generally emerges in longer-term paradigms and in models with progressive axonal degeneration, which contrasts with the early inflammatory phase examined in our short-term CitiQ10 protocol.

Although our results did not show a statistically significant effect of CitiQ10 treatment on RNFL thickness, treated eyes exhibited less pronounced thickening than untreated ones, likely due to reduced glial activation. To date, no animal studies have evaluated isolated or CitiQ10 treatment on RNFL thickness. However, clinical studies suggest that citicoline may stabilize RNFL thickness in glaucoma patients, with placebo groups showing progressive thinning [74,75]. Combinations of citicoline with homotaurine and vitamin E have also demonstrated structural stability [76]. Topical treatment with CoQ10 in patients with glaucoma stabilized RNFL thickness [77]. Moreover, in patients with Alzheimer’s disease, topical treatment with CoQ10 prevented RNFL thinning [78].

### 3.2. Effect of Citicoline and CoQ10 on Vitreous Inflammatory Particles Following OHT Induction

This study evaluated the presence of vitreous particles in OCT images near the optic nerve head as a potential indirect marker of macrophage-related inflammatory activity. Assessments were performed at 3 and 7 days post-induction, revealing a significant increase in particle count in OHT-untreated eyes compared to controls at both time points. Although no prior studies have examined this variable in the same laser-induced OHT model, similar findings have been reported in chronic glaucoma models using microsphere injections, where increased vitreous particles were observed during inflammatory phases [14,18].

In our model, the rise in vitreous particles at 3 days post-induction coincided with peak glial activation, as previously described by our group using the same model [37,56,58]. This temporal overlap suggests that the increased particle presence may be directly linked to retinal inflammation triggered by elevated IOP. In our model, the increase in vitreous particles at 3 days post-induction coincided with the peak of glial activation previously reported in this experimental paradigm [28,44,45]. This temporal overlap suggests that the rise in particle number may be associated with retinal inflammation triggered by elevated IOP. It is important to note, however, that OCT-based detection of hyperreflective vitreous particles does not allow definitive identification of their cellular nature. Therefore, their interpretation as inflammatory cells is indirect and based on their temporal association with glial activation and on previous histological evidence from similar models [37,42,58,60]. This interpretation is further supported by recent findings in a steroid-induced glaucoma model, where OCT-detected vitreous hyperreflective particles corresponded to immune cells infiltrating the vitreous chamber [14]. The morphology and distribution of the particles described in the study of Rodrigo et al. [14] closely resemble those observed here, reinforcing the plausibility of an inflammatory origin. Nonetheless, because OCT cannot directly determine cellular identity, this interpretation should be regarded as inferential.

### 3.3. Effect of Citicoline and CoQ10 on Retinal Electrical Activity Following OHT Induction

Although previous studies have primarily linked ERG alterations to later stages of OHT, our findings reveal that significant reductions in scotopic b-wave, mixed b-wave, and OPs occur as early as three days post-induction. At 7 days post-induction, additional impairments were observed in mixed a-wave, photopic b-wave, and flicker responses, indicating progressive functional deterioration. These results align with previous work in the same model, which reported ERG amplitude loss within 24 h of OHT induction, likely due to acute axonal transport disruption, axotomy-like degeneration, and structural damage to RGCs and bipolar and photoreceptor cells [27]. Additionally, mechanical compression of the optic nerve head may compromise retinal blood supply, particularly to inner layers [79].

At both 3 and 7 days post-induction, contralateral eyes showed a trend toward reduced wave amplitudes, although this did not reach statistical significance. This subtle decline may suggest that these eyes begin to exhibit early functional changes, potentially influenced by the pathological alterations occurring in the OHT eye. Importantly, these mild and non-significant functional tendencies are consistent with the absence of RGC loss previously reported [27] in contralateral eyes at the same time points. Although early inflammatory signaling has been described histologically in this model, it does not appear to result in measurable electrophysiological deficits. Overall, these findings indicate that contralateral retinal function remains largely preserved during the early phase of unilateral OHT.

Neuroinflammation may also contribute to early ERG decline. At day three, intense glial activation is evident in this model [56,58], and microglial activation in neurodegenerative contexts is known to trigger pro-inflammatory cytokine release and complement system activation, leading to synaptic pruning even before neuronal death [80]. This excessive pruning reduces synaptic plasticity and disrupts neural networks. In previous studies using our model, rod-shaped microglia were observed along RGC axons, closely interacting with somas and dendrites, suggesting active synapse elimination during early inflammation [63].

The temporal pattern of ERG decline suggests initial impairment of scotopic responses (b-wave, mixed b-wave, and OPs) followed by photopic deficits at 7 days post-induction. ERG techniques can differentiate ON and OFF pathways. For example, in ffERG, long-duration photopic flashes elicit b-waves (ON) and d/i-waves (OFF). Flicker stimuli show low-frequency responses linked to ON and high-frequency responses to OFF pathways [81]. In glaucoma models, high-frequency flicker responses are more affected [82], a finding confirmed in clinical studies where temporal frequencies between 15 and 50 Hz are most compromised in glaucomatous eyes.

Despite these insights, no statistically significant differences were found between treated and untreated OHT eyes in any ERG parameter at either time point. However, treated eyes consistently showed higher ERG amplitudes, suggesting a potential tendency toward functional preservation.

To date, no studies have evaluated the combined effects of citicoline and CoQ10 using the same electrophysiological approach. Nonetheless, clinical research has demonstrated that citicoline improves visual function in glaucoma patients, with benefits observed in pattern ERG and VEP [48,83,84,85]. In kainic acid-induced retinal injury models, citicoline preserved both inner and outer retinal layers, indicating functional neuroprotection [86,87]. CoQ10 has shown similar benefits: in ischemia–reperfusion models, it improved a- and b-wave amplitudes and in oxidative stress models, it enhanced VEP responses [52,88]. Topical CoQ10 with vitamin E improved visual and cortical function in glaucoma patients [77,89]. Although most of these studies focus on RGC and cortical function, they collectively support the notion that citicoline and CoQ10 may contribute to broader visual preservation.

Several experimental glaucoma therapies have also been evaluated functionally using ERG, although most have been conducted in rats and rely on hypertensive models and analysis timelines that differ substantially from ours. Subconjunctival erythropoietin in a limbal photocoagulation rat model showed partial recovery of photopic responses by day 21 [90], while the mitochondria-targeted peptide SS 31 improved a- and b-wave amplitudes only after several weeks in a microbead-induced model [91]. In contrast, spearmint extract supplementation did not modify ERG responses in a methylcellulose-based hypertensive rat model [92], whereas methylene blue preserved scotopic amplitudes at 15 days in an episcleral cauterization model [93]. Herbal eye drops containing *Rosmarinus officinalis*, *Foeniculum vulgare*, and *Helichrysum italicum* also maintained ERG amplitudes at 4–8 weeks in microbead-induced glaucoma in rats [94]. Taken together, these studies indicate that functional rescue is typically observed at later stages or under treatments with strong mitochondrial or antioxidative actions, which is consistent with the absence of early ERG alterations in our short-term CitiQ10 protocol in mice. Longer follow-up will be required to determine whether the higher ERG amplitudes observed in treated eyes translate into sustained functional protection as inflammation resolves and RGC loss progresses.

### 3.4. Translational Relevance of Citicoline and CoQ10 Neuroprotection

Taken together, the structural protection observed by OCT and the trends toward higher ERG amplitudes in CitiQ10-treated OHT eyes suggest that these compounds may influence early retinal dysfunction at multiple levels. Although electrophysiological differences did not reach statistical significance, the parallel changes observed in retinal morphology raise relevant questions about their potential translational value in human glaucoma.

To better contextualize these findings, it is important to consider the complementary mechanisms through which both compounds may exert neuroprotection. The potential actions of citicoline and CoQ10 in early OHT can be interpreted through three interacting mechanisms. CoQ10 primarily supports mitochondrial resilience by maintaining electron transport efficiency, limiting ROS production, and reducing pro-apoptotic signaling, such as Bax activation, while favoring survival-associated proteins like pBad and Bcl-xL [52,95,96,97]. It also preserves mitochondrial DNA and OXPHOS components, protects against glutamate-induced toxicity and oxidative stress, enhances respiratory capacity through its reduced form ubiquinol [52,95,97], improves glutamate transporter function, and activates protective pathways such as PKB/Akt [97,98,99]. Citicoline, in turn, supports membrane integrity and synaptic stability by promoting phosphatidylcholine synthesis and facilitating the repair of stressed neuronal membranes [100]. Clinical and experimental studies have associated citicoline with improvements in visual function, enhanced neurotransmission, and reduced glutamate accumulation through increased astrocytic transporter expression [48,49,74,75,83,84,85,86,101,102,103,104,105,106,107,108,109,110,111]. Finally, both compounds may also influence early glial responses, as suggested by a previous study from our group reporting reduced microglial activation and GFAP expression in similar experimental conditions [37]. Together, these mitochondrial, membrane-stabilizing, and glial-modulating actions may converge to mitigate the metabolic and inflammatory stress triggered by acute IOP elevation.

In clinical practice, citicoline and CoQ10 have been used separately as neuroprotective supplements in glaucoma. Citicoline has shown beneficial effects on retinal structure and function in patients with primary open-angle glaucoma with controlled IOP, across different routes of administration. Early studies using intramuscular citicoline reported improvements in visual fields [47]. Subsequent topical formulations enhanced electrophysiological responses, including pattern ERG and visual evoked potentials, and were associated with improvements in visual fields and RNFL thickness measured by OCT [48,107,109]. Oral citicoline (250–600 mg/day) has also been shown to improve or stabilize visual fields, RNFL thickness, VEP parameters [49,74,75,103,112], and neural conduction, reducing retinocortical time and suggesting synaptic plasticity in post-retinal pathways [85].

Clinical studies evaluating CoQ10 in glaucoma are fewer, but topical administration of CoQ10 combined with vitamin E has demonstrated improvements in electrophysiological responses [77,89] and maintained RNFL thickness [77] in open-angle glaucoma and reduced oxidative stress in aqueous humor samples from pseudoexfoliative glaucoma patients [113]. Importantly, recent pharmacokinetic evidence indicates that novel oral timed-release formulations of CoQ10 achieve improved bioavailability and sustained plasma concentration in healthy subjects [114], supporting its potential for systemic therapeutic applications. To date, no clinical studies have assessed the combined use of citicoline and CoQ10, highlighting the novelty and translational interest of our experimental findings.

Together, these clinical observations mirror the early structural and electrophysiological changes observed in our model and further support the relevance of targeting early inflammatory and metabolic alterations as a therapeutic strategy in glaucoma management.

### 3.5. Study Limitations

A limitation of the present study is that only the combined formulation of citicoline and CoQ10 was evaluated. Although this choice reflects the preparation currently used in clinical practice, it prevents us from determining the individual contribution of each compound or distinguishing additive from synergistic interactions. Previous experimental evidence indicates that both molecules exert neuroprotective effects when administered separately, and that their combined use may further reduce oxidative stress, inflammatory mediators, and pro-apoptotic signaling in retinal and glial models, suggesting potential cumulative or synergistic actions [43,51,53,115,116]. Additionally, because the present study examines only very early time points (3 and 7 days post-induction), no long-term structural or functional preservation can be inferred. Future studies including single-compound treatment arms, dose–response analyses, extended follow-up periods, and direct comparisons with the combined formulation will be necessary to clarify the relative contribution of each molecule and to better characterize potential mechanistic interactions within this neuroprotective strategy.

## 4. Materials and Methods

### 4.1. Animals

This study adhered to institutional policies, European Union directives concerning the use of animals in scientific research, and the ARVO (Association for Research in Vision and Ophthalmology) Statement for the Use of Animals in Ophthalmic and Vision Research. Furthermore, the manuscript was prepared in accordance with the ARRIVE guidelines (Animal Research: Reporting of In Vivo Experiments), which provide recommendations for the transparent reporting of animal-based research.

All procedures were carried out in strict compliance with ethical standards established by Spanish legislation and the Guidelines for Humane Endpoints for Animals Used in Biomedical Research. The experimental protocol received approval from the Animal Research Ethics Committee of Complutense University (Madrid, Spain) and the General Directorate of Agriculture and Food of the Ministry of Economy and Employment of the Community of Madrid (PROEX 091.2/22).

The study involved 60 adult male CD-1 Swiss albino mice, aged between 12 and 16 weeks and weighing 35–45 g. The animals were obtained from Charles River Laboratory (Barcelona, Spain) and housed in the animal care facility of the School of Medicine at Complutense University of Madrid (Madrid, Spain). Throughout the experimental period, the mice were kept under standardized environmental conditions, including a 12 h light/dark cycle (light intensity: 9–24 lux) and regulated temperature. They had unrestricted access to both standard rodent chow and water.

### 4.2. Experimental Groups

The mice were randomly assigned to one of four experimental conditions:

1. Control vehicle (vehicle) group (*n* = 12), serving as the negative control, received neutral gelatin throughout the study without undergoing any additional interventions.
2. Control CitiQ10 group (CitiQ10) (*n* = 12), serving as the positive control, was administered citicoline and CoQ10 for the entire duration of the experiment, without undergoing any procedures.
3. OHT group treated with vehicle (OHT) (*n* = 18), consisting of mice that received neutral gelatin and were subjected to laser-induced elevation of IOP. The group was subdivided such that the left eye was designated as the OHT eye and the right eye as the Contralateral-OHT eye.
4. OHT group treated with CitiQ10 (OHT-CitiQ10) (*n* = 18), in which animals were exposed to laser-induced OHT and simultaneously treated with citicoline and CoQ10. The group was subdivided such that the left eye was designated as the OHT-CitiQ10 eye and the right eye as the contralateral OHT-CitiQ10 eye.

For both groups undergoing OHT induction (OHT and OHT-CitiQ10), evaluations were conducted on the lasered eye (left eye) and the untreated contralateral eye (right eye) at two time points: on 3 and 7 days post-induction.

### 4.3. Administration Protocol for Citicoline and Coenzyme Q10

**Figure 10:**
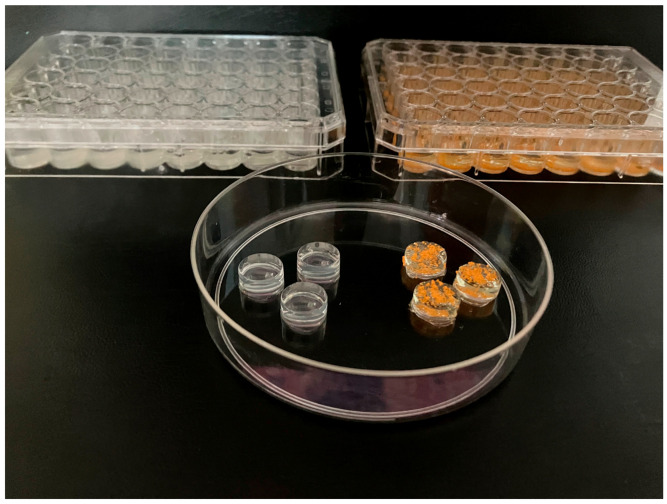
Representative samples of the two types of gelatine that were placed in the multiwell plates, along with one capsule of each formulation. The transparent gelatine corresponds to the neutral formulation administered to the vehicle groups, while the orange-colored gelatine contains the combination of citicoline and coenzyme Q10 administered to the CitiQ10 groups.

The administration of CitiQ10 followed the methodology previously described (Matamoros et al., 2024; 2025) [36,37]. Briefly, animals underwent a one-week training period with daily administration of plain gelatin to familiarize them with the oral delivery method, followed by the initiation of treatment 15 days prior to the induction of OHT, which was maintained daily until animal sacrifice at 3 or 7 days post-induction. Each day, the animals received an oral dose of 0.5 mL of gelatine, prepared in our laboratory using drinking water and porcine gelatine (Sigma-Aldrich G2500-500G, Merck KGaA, Darmstadt, Germany). For the treatment groups, the gelatine was enriched with citicoline (Neurotidine^®^, Omikron Pharmaceutical España S.L.U., Barcelona, Spain) at a concentration of 500 mg/kg and coenzyme Q10 (COQUN^®^ OS, VISUfarma B.V., Madrid, Spain) at 200 mg/kg. This therapeutic combination is commercially available under the name COQUN^®^ Combo (VISUfarma S.p.A., Rome, Italy). Dosage calculations were based on an estimated average body weight of 40 g per mouse, as outlined in the referenced protocol (Figure 10) [36].

Animals assigned to the control vehicle group received gelatine without active compounds. Administration began 15 days prior to OHT induction and continued until the animals were sacrificed at either 3 or 7 days post-induction.

To promote consistent ingestion and minimize variability, all mice underwent a one-week acclimatization period during which they received daily 0.5 mL portions of plain gelatin, prepared with potable water and the same porcine gelatine source, prior to the start of the experimental treatment. This pre-conditioning phase ensured that the animals were familiar with the delivery method and consistently consumed the gelatine matrix throughout the study.

### 4.4. Anesthetic Procedures

Anesthesia protocols were implemented in accordance with previously established methodologies (Matamoros et al., 2024) [36]. For all surgical interventions—including OHT induction and euthanasia—mice were anesthetized using an intraperitoneal combination of ketamine (Anestekin^®^, 100 mg/mL; Dechra Veterinary Products SLU, Barcelona, Spain), medetomidine (Dormisan^®^, 1 mg/mL; Fatro Ibérica, Barcelona, Spain), and sterile saline solution.

To facilitate post-anesthetic recovery, a subcutaneous injection of 0.1 mL atipamezole hydrochloride (Nosedorm^®^, 5 mg/mL; Laboratorios Karizoo S.A., Barcelona, Spain) was administered following the procedures. Prior to laser application for OHT induction, topical anesthesia was applied to the corneal surface using a dual-action formulation containing tetracaine hydrochloride (1 mg/mL) and oxybuprocaine hydrochloride (4 mg/mL), marketed as COLICURSÍ™ ANESTÉSICO DOBLE (Alcon España, Barcelona, Spain).

IOP assessments were performed under inhalational anesthesia, using a mixture of 2% isoflurane in oxygen (Isoflutek^®^, 1000 mg/g; Laboratorios Karizoo S.A., Barcelona, Spain), ensuring minimal distress and accurate measurement conditions.

### 4.5. Procedure for Inducing Ocular Hypertension and Monitoring Intraocular Pressure

To induce OHT, we employed the laser photocoagulation protocol described previously [35], using the Viridis Ophthalmic Photocoagulator (532 nm; Quantel Medical, Clermont-Ferrand, France). Laser application targeted the limbal and episcleral veins, with the following parameters: 50 µm spot diameter, 0.3 W power output, and 0.5 s exposure per pulse. Each mouse received between 80 and 150 laser pulses.

Immediately after the laser procedure, a topical ophthalmic solution containing dexamethasone (1 mg/mL) and tobramycin (3 mg/mL) (Tobradex^®^, Alcon, Barcelona, Spain) was applied to the treated eye to mitigate risks of corneal irritation, inflammation, or infection.

IOP was assessed following the anesthesia protocol previously outlined, using a rebound tonometer (TonoLab; Tiolat, OY, Helsinki, Finland), as described in previous studies [35,42,56]. Measurements were taken from the lasered (OHT) eye and the contralateral eye across all experimental groups. For each time point, IOP was calculated as the mean of three separate readings, with each reading representing the automated average of six consecutive measurements.

Baseline IOP values were recorded prior to laser OHT induction. Post-induction, IOP was monitored at 5 distinct intervals: 24 h, 48 h, and on days 3, 5, and 7. All measurements were consistently performed at 10:00 AM to control fluctuations due to circadian variation.

### 4.6. ffERG Protocol

Prior to electrophysiological testing, mice were kept in complete darkness for 24 h to ensure full scotopic adaptation. All handling during this period was performed under dim red illumination to preserve their dark-adapted state. Once anesthetized, animals were placed inside a Faraday box to shield them from ambient electrical interference and positioned on an electric blanket with thermostatic control (T/Pump TPP522, Gaymar Industries, Orchard Park, NY, USA) to maintain core body temperature at 37 °C.

To ensure consistent retinal exposure to light stimuli, pupil dilation was achieved using a topical application of 1% tropicamide (Alcon Cusí, SA, El Masnou, Barcelona, Spain). For signal acquisition, a subcutaneous needle was inserted at the tail base to serve as the ground electrode, while a gold reference electrode was positioned on the tongue. Retinal activity was recorded via a fine gold ring electrode placed directly on the corneal surface. To enhance electrical conductivity and prevent corneal dehydration, a drop of 2% methylcellulose (Methocel, Omnivision, Neuhausen, Switzerland) was applied previously to the gold ring micropositioning.

Recordings were conducted monocularly using full-field light stimuli generated within a Ganzfeld dome, which provides uniform retinal illumination. Scotopic responses were elicited using a series of flash intensities ranging from −4.0 to 1.5 log cd·s·m^−2^. The retinal signals were amplified and filtered within a frequency range of 0.3 to 1000 Hz using a CP511 AC amplifier (Grass Instruments, Quincy, MA, USA). Oscillatory potentials were specifically recorded following stimulation at 1.5 log cd·s·m^−2^, using a narrower bandpass filter between 30 and 10,000 Hz.

Following the scotopic phase, animals were exposed to a background light of 30 cd·m^−2^ for 5 min to induce photopic adaptation. Subsequent photopic recordings were obtained using flash intensities from −1.0 to 1.5 log cd·s·m^−2^, with signals filtered between 0.3 and 1000 Hz. Flicker responses were captured at a frequency of 20 Hz and intensity of 1.5 log cd·s^−1^·m^−2^.

All electrophysiological data were digitized at a sampling rate of 20 kHz using a Power Lab 4/35 acquisition system (ADInstruments Ltd., Oxford, UK) and were visualized on a computer. Waveform components of the ERG were manually analyzed using LabChart Pro software version 8.1.13 (ADInstruments Ltd., Oxford, UK).

### 4.7. OCT Protocol

All animals had in vivo retinal imaging using SD-OCT. Before scanning, the pupils were dilated with a drop of 10 mg/mL tropicamide (Colircusí Tropicamide, Alcon Healthcare, Barcelona, Spain). Images were obtained using the Spectralis SD-OCT system, equipped with Heidelberg Eye Explorer software for animal studies (version 7.0.4; Heidelberg Engineering, Heidelberg, Germany). To maintain physiological temperature during the procedure, the animals were placed on heating pads. Corneal hydration was preserved by applying a drop of cross-linked sodium hyaluronate (VisuXL^®^, Visufarma SpA, Rome, Italy).

To accommodate the anatomical differences between mouse and human eyes, a +25-dioptre lens was mounted on the OCT device to achieve adequate retinal focus. In addition, a rigid contact lens (3.2 mm diameter and 1.7 base curvature; F2 LOW, VERAPERM VET, Interlenco, Madrid, Spain) was placed on the mouse’s eye to standardize the corneal surface and prevent desiccation during the procedure.

Image acquisition was centered on the optic nerve head (ONH) and real-time tracking was enabled to minimize motion artifacts caused by breathing or involuntary eye movements.

#### 4.7.1. Retinal Thickness Evaluation

**Figure 11:**
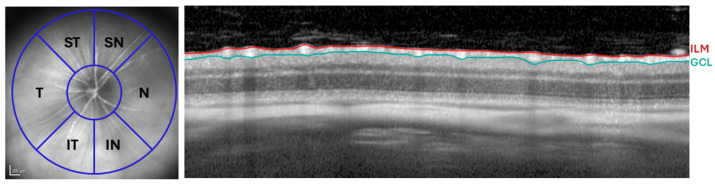
RNFL analysis by OCT. Distribution of the RNFL sectors: SN, ST, N, T, IN, and IT (**Left**). OCT section showing the thickness of the RNFL, delimited between the ILM and the GCL (**Right**). Abbreviations: RNFL (retinal nerve fiber layer), ILM (inner limiting membrane), GCL (ganglion cell layer), OCT (optical coherence tomography), N (nasal), T (temporal), SN (supero-nasal), ST (supero-temporal), IN (infero-nasal), and IT (infero-temporal).

Quantitative analysis of retinal thickness was carried out following the peripapillary segmentation as described previously [117]. For the RNFL thickness assessment, six anatomical sectors were examined: inferior-nasal (IN), nasal (N), superior-nasal (SN), superior-temporal (ST), temporal (T), and inferior-temporal (IT) (Figure 11). All retinal segmentations measured were manually performed by the same experienced researcher.

#### 4.7.2. Vitreous Particle Quantification

To identify and quantify particulate matter within the vitreous body, image analysis was performed using Fiji (ImageJ) (https://imagej.net/software/fiji/ accessed on 18 January 2026). Cross-sectional scans intersecting the center of the optic nerve were selected from the Spectralis OCT2 system (Heidelberg Engineering, Heidelberg, Germany). Images were exported and converted to 16-bit grayscale to enhance contrast resolution.

**Figure 12:**
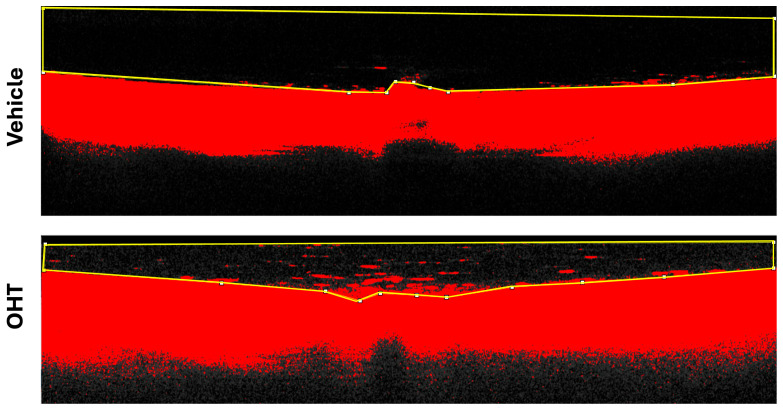
Fiji analysis of vitreous particles. OCT image of a retinal section corresponding to the optic nerve head region, with the selected ROI for measurement. Abbreviations: OHT (ocular hypertension); OCT (optical coherence tomography).

A manual threshold adjustment was applied to isolate vitreous particles from background noise. Once the optimal threshold was established, a region of interest (ROI) encompassing the relevant vitreous area was defined. Particle quantification was then conducted using the “Analyze Particles” function in Fiji, allowing for precise enumeration of particles per image (Figure 12).

Vitreous hyperreflective particles were quantified manually by two independent observers masked to the experimental groups. To ensure consistency, both observers followed the same predefined counting criteria, and a subset of images was evaluated by both to confirm agreement in particle identification and quantification. In addition, one observer repeated the analysis in a subset of images to verify intra-observer consistency. These procedures support the robustness and reliability of the particle quantification method.

### 4.8. Statistical Evaluation

All statistical procedures were conducted using GraphPad Prism software, version 9 (GraphPad Software, La Jolla, CA, USA). To assess differences across experimental conditions, a two-way analysis of variance (ANOVA) was applied, followed by Tukey’s post hoc test for multiple comparisons.

Data are presented as mean values ± standard deviation (SD). Statistical significance was defined as *p* < 0.05. The following symbols were used to indicate varying degrees of significance: *p* < 0.05 (*), *p* < 0.01 (**), *p* < 0.001 (***), and *p* < 0.0001 (****).

The association between the maximum intraocular pressure (IOP) and the number of vitreous particles, assuming a Gaussian distribution, was evaluated using Pearson’s correlation coefficient (r). Scatter plots were used to visualize the null hypothesis for pairwise correlations, with unadjusted *p*-values indicating the strength and direction of the linear relationship between the variables.

## 5. Conclusions

This study demonstrates that CitiQ10 treatment may exert both anti-inflammatory and neuroprotective effects in a laser-induced OHT model in albino Swiss mice. Structural analysis using OCT revealed early thickening of the RNFL in OHT eyes, likely associated with glial activation. This thickening was less pronounced in treated eyes, suggesting a modulatory effect of the therapy on retinal inflammation. The presence of hyperreflective particles in the vitreous, considered indirect markers of inflammatory activity, was significantly reduced in treated eyes, further supporting the anti-inflammatory potential of the combination.

Functional assessment via ERG showed early and progressive impairment of retinal responses following OHT induction. Although treated eyes exhibited slightly higher ERG amplitudes than untreated eyes, these differences did not reach statistical significance and should therefore be interpreted as trends rather than evidence of functional preservation. Together with the structural findings, these results suggest that citicoline and CoQ10 may influence early retinal alterations. Further investigation is needed to clarify the clinical relevance of these early findings and to explore the potential utility of this combination in glaucoma management.

## Data Availability

The data presented in this study are available on request from the corresponding author. The data are not publicly available due to patentability concerns.

## Abbreviations

The following abbreviations are used in this manuscript:

CitiQ10	Citicoline + Coenzyme Q10 treatment
CoQ10	Coenzyme Q10
ERG	Electroretinography
ffERG	Full-Field Electroretinography
GCC	Ganglion Cell Complex
GFAP	Glial Fibrillary Acidic Protein
I	Inferior
IN	Inferior-Nasal
INL	Inner Nuclear Layer
IOP	Intraocular Pressure
IT	Inferior-Temporal
N	Nasal
OCT	Optical Coherence Tomography
OHT	Ocular Hypertension
ONL	Outer Nuclear Layer
OPs	Oscillatory Potentials
PhNR	Photopic Negative Response
RGCs	Retinal Ganglion Cells
RNFL	Retinal Nerve Fiber Layer
SD-OCT	Spectral Domain Optical Coherence Tomography
SN	Superior-Nasal
ST	Superior-Temporal
STR	Scotopic Threshold Response
T	Temporal
VEP	Visual Evoked Potential
